# Data on attitudes, religious perspectives, and practices towards COVID-19 among Indonesian residents: a quick online cross-sectional survey

**DOI:** 10.1016/j.dib.2020.106277

**Published:** 2020-09-05

**Authors:** Zulvikar Syambani Ulhaq, Risma Aprinda Kristanti, Achmad Arief Hidayatullah, Lailia Nur Rachma, Nurlaili Susanti, Aulanni'am Aulanni'am

**Affiliations:** 1 Faculty of Medicine and Health Sciences, Maulana Malik Ibrahim State Islamic University of Malang, Batu, Indonesia; 2 Faculty of Science, Brawijaya University, Malang, Indonesia

**Keywords:** Survey data, Online questionnaire, Cross-sectional, COVID-19, Indonesian residents, Attitude, Religious perspective, Practice

## Abstract

Although previously large-scale social restrictions were implemented by the Indonesian government, the total number of coronavirus cases is overcome China in the global ranking per July 18th, 2020, implying a higher infection rate among Indonesian residents. The surge of new coronavirus cases started since the loosening of large-scale social restrictions, thereby implicating that public gathering (including religious gathering) evidently increases transmission [1]. It has been reported that Indonesia's coronavirus disease-19 (COVID-19) mortality rate is the second-highest among Southeast Asian Nations, which may be associated with several health determinants, including biochemical factors and health comorbidity [2], [3], [4], [5], [6], [7]. Because people's adherence to control measures is affected by their attitudes, religious perspectives, and practices (ARP) towards COVID-19. Hence, the information regarding Indonesian's ARP towards COVID-19 post-large-scale social restrictions is required. The data were collected via an online questionnaire, including demographic information (7 items), attitude and practice (5 items), and religious perspective and practice (5 items), from July 11 – 18, 2020, collecting a total of 1,345 respondents. Although our data collection did not provide other precautionary measures (e.g., adequate ventilation). It is notable that most of the religious venues are having a close ventilation system. Hence, this may contribute to the propagation of SARS-CoV-2 transmission [8]. Altogether, these data will help in determining non-health-related factors to prevent the spread of COVID-19.

## Specifications Table

**Table:** 

Subject	Public health
Specific subject area	Health psychology, Social psychology
Type of data	Primary data, tables
How data were acquired	Data were collated utilizing an online survey platform (Google forms)
Data format	Raw and analyzed
Parameters for data collection	The survey data was obtained from 1,354 respondents of Indonesian residents with internet access.
Description of data collection	The data was conducted through an online questionnaire. Relying on the authors’ network, one-page recruitment information was posted/reposted via Whatsapp.
Data source location	Region: Asia; Country: Indonesia
Data accessibility	Data is accessible at Mendeley Repository https://data.mendeley.com/datasets/nswtwm7j8k/1

## Value of Data

• This data describes the attitude, religious perspective, and practice among Indonesian residents toward COVID-19.
• This data is useful for researchers who want to compare similar studies regarding attitude, religious perspective, and practice toward COVID-19 in the different populations.
• This data may help the leaders and policymakers to evaluate and prevent non-health-related factors associated with the spread of COVID-19.

## 1 Data Description

A total of 1,354 participants completed the questionnaire on attitude, religious perspective, and practice among Indonesian residents toward COVID-19 (Table 1), which then was divided according to demographic characteristics (Table 2). The detailed responses on attitude, religious perspective, and practice toward COVID-19 by participants are presented in Table 3–4. Factors associated with attitude, religious perspective, and practice toward COVID-19 are depicted in Table 5.

**Table 1:** Questionnaire of attitude, religious perspective, and practice towards COVID-19

Questions	Options
Attitude and practice	
A1. Do you think physical distancing effectively cut the spread of SARS-CoV-2 infection?	Yes, not sure, no
A2. Do you feel anxious about getting infected with SARS-CoV-2?	Yes, not sure, no
A3. Do you think it is necessary to perform more radical control such as “comprehensive large-scale social restriction or lockdown” one more time as the new cases growing rapidly in the last few days?	Yes, not sure, no
AP1. Have you applied health protocol regarding COVID-19 prevention (worn mask, washed hands) when you were outside home and in a crowded place?	Always, occasionally, never
AP2. Have you applied health protocol regarding COVID-19 prevention (worn mask, washed hands) when you were home after traveling/working outside?	Always, occasionally, never
Religious perspective and practice	
R1. Do you think it is possible to pray and gather in the place of worship during the pandemic?	Yes, not sure, no
R2. Are the place of worship conducted COVID-19 prevention control correctly?	Yes, not sure, no
R3. Does social distancing in the place of worship make you feel safe from SARS-CoV-2 infection?	Yes, not sure, no
RP1. In the recent days, have you prayed in the place of worship other than in your home?	Yes, no
RP2. How often did you pray and gather in the place of worship during “new normal or post large-scale social restrictions” was implemented?	Always, occasionally, never

**Table 2:** Demographic characteristics of participants (n = 1,354)

Characteristics		Number of participants (%)
Gender	Male	368 (27.18)
	Female	986 (72.82)
Age group (years)	< 30	939 (69.35)
	≥ 30	415 (30.65)
Last education	High school	437 (32.27)
	Associate degree	222 (16.40)
	Bachelor degree	499 (36.85)
	Master degree	149 (11.00)
	Doctoral degree	47 (3.48)
Majors of current education or major of education	Medicine related science	1,044 (77.10)
	Science and technology	195 (14.40)
	Social science and humanities	115 (8.50)
Occupation	Students	664 (49.03)
	Teachers	168 (12.41)
	Health practitioners	382 (28.21)
	Government and administration related job	33 (2.89)
	Others	107 (7.90)
Religion	Islam	1,167 (86.19)
	Protestantism	89 (6.57)
	Roman Catholicism	55 (4.06)
	Hinduism	33 (2.44)
	Buddhism	9 (0.66)
	Confucianism	1 (0.08)
Place of current residence	City	943 (69.65)
	Rural	411 (30.35)

**Table 3:** Attitude and practice towards COVID-19 by demographic variables*

Characteristic		Attitudes and practice, n (%)														
		A1: physical distancing cut the spread of SARS-CoV-2			A2: feeling anxious being infected with SARS-CoV-2			A3: large-scale social restriction need to be re-implemented			AP1: COVID-19 prevention in a crowded place			AP2: COVID-19 prevention after traveling or working outside		
		Y	NS	N	Y	NS	N	Y	NS	N	A	O	NV	A	O	NV
Gender	Male	338	27	3	262	57	49	268	66	34	320	35	13	323	36	9
		(91.85)	(7.34)	(0.81)	(71.20)	(15.49)	(13.31)	(72.83)	(17.93)	(9.24)	(86.96)	(9.51)	(3.53)	(87.77)	(9.78)	(2.45)
	Female	931	51	4	773	135	78	775	165	46	926	46	14	935	42	9
		(94.42)	(5.17)	(0.41)	(78.40)	(13.69)	(7.91)	(78.60)	(16.73)	(4.67)	(93.91)	(4.67)	(1.42)	(94.83)	(4.26)	(0.91)
Age group (years)	< 30	878	59	2	712	146	81	708	178	53	860	57	22	875	53	11
		(93.50)	(6.28)	(0.22)	(75.83)	(15.55)	(8.62)	(75.40)	(18.96)	(5.64)	(91.59)	(6.07)	(2.34)	(93.18)	(5.65)	(1.17)
	≥ 30	391	19	5	323	46	46	335	53	27	386	24	5	383	25	7
		(94.22)	(4.58)	(1.20)	(77.84)	(11.08)	(11.08)	(80.72)	(12.77)	(6.51)	(93.02)	(5.78)	(1.20)	(92.29)	(6.02)	(1.69)
Last education	High school	401	33	3	325	76	36	325	85	27	403	24	10	410	24	3
		(91.76)	(7.55)	(0.69)	(74.37)	(17.39)	(8.24)	(74.37)	(19.45)	(6.18)	(92.22)	(5.49)	(2.29)	(93.82)	(5.49)	(0.69)
	Associate degree	218	4	0	176	22	24	196	17	9	197	16	9	202	12	8
		(98.20)	(1.80)	(0.00)	(79.28)	(9.91)	(10.81)	(88.29)	(7.66)	(4.05)	(88.74)	(7.21)	(4.05)	(90.99)	(5.41)	(3.60)
	Bachelor degree	469	29	1	391	61	47	372	100	27	462	31	6	465	29	5
		(93.99)	(5.81)	(0.20)	(78.36)	(12.22)	(9.42)	(74.55)	(20.04)	(5.41)	(92.59)	(6.21)	(1.20)	(93.19)	(5.81)	(1.00)
	Master degree	136	11	2	110	24	15	119	19	11	141	7	1	139	8	2
		(91.28)	(7.38)	(1.34)	(73.83)	(16.11)	(10.07)	(79.87)	(12.75)	(7.38)	(94.63)	(4.70)	(0.67)	(93.29)	(5.37)	(1.34)
	Doctoral degree	45	1	1	33	9	5	31	10	6	43	3	1	42	5	0
		(95.74)	(2.13)	(2.13)	(70.21)	(19.15)	(10.64)	(65.96)	(21.28)	(12.77)	(91.49)	(6.38)	(2.13)	(89.36)	(10.64)	(0.00)
Major of education	Medicine related science	987	52	5	795	148	101	824	166	54	963	58	23	981	48	15
		(94.54)	(4.98)	(0.48)	(76.15)	(14.18)	(9.67)	(78.93)	(15.90)	(5.17)	(92.24)	(5.56)	(2.20)	(93.97)	(4.60)	(1.44)
	Science and technology	178	17	0	153	33	9	137	41	17	176	16	3	171	21	3
		(91.28)	(8.72)	(0.00)	(78.46)	(16.92)	(4.62)	(70.26)	(21.03)	(8.72)	(90.26)	(8.21)	(1.54)	(87.69)	(10.77)	(1.54)
	Social science and humanities	104	9	2	87	11	17	82	24	9	107	7	1	106	9	0
		(90.43)	(7.83)	(1.74)	(75.65)	(9.57)	(14.78)	(71.30)	(20.87)	(7.83)	(93.04)	(6.09)	(0.87)	(92.17)	(7.83)	(0.00)
Occupation	Students	617	45	2	498	117	49	480	146	38	607	42	15	617	40	7
		(92.92)	(6.78)	(0.30)	(75.00)	(17.62)	(7.38)	(72.29)	(21.99)	(5.72)	(91.41)	(6.33)	(2.26)	(92.92)	(6.02)	(1.06)
	Teachers	158	7	3	128	23	17	125	30	13	157	9	2	152	13	3
		(94.05)	(4.17)	(1.79)	(76.19)	(13.69)	(10.12)	(74.40)	(17.86)	(7.74)	(93.45)	(5.36)	(1.19)	(90.48)	(7.74)	(1.79)
	Health practitioners	370	10	2	298	38	46	328	35	19	354	22	6	358	18	6
		(96.86)	(2.62)	(0.52)	(78.01)	(9.95)	(12.04)	(85.87)	(9.16)	(4.97)	(92.67)	(5.76)	(1.57)	(93.72)	(4.71)	(1.57)
	Government and administration related job	33	0	0	28	2	3	30	2	1	29	2	2	31	2	0
		(100)	(0.00)	(0.00)	(84.85)	(6.06)	(9.09)	(90.91)	(6.06)	(3.03)	(87.88)	(6.06)	(6.06)	(93.94)	(6.06)	(0.00)
	Others	91	16	0	83	12	12	80	18	9	99	6	2	100	5	2
		(85.05)	(14.95)	(0.00)	(77.58)	(11.21)	(11.21)	(74.77)	(16.82)	(8.41)	(92.52)	(5.61)	(1.87)	(93.46)	(4.67)	(1.87)
Place of current residence	City	881	55	7	746	116	81	721	153	60	877	48	18	885	48	10
		(93.43)	(5.83)	(0.74)	(79.11)	(12.30)	(8.59)	(77.19)	(16.38)	(6.42)	(93.00)	(5.09)	(1.91)	(93.85)	(5.09)	(1.06)
	Rural	388	23	0	289	76	46	313	78	20	369	33	9	373	30	8
		(94.40)	(5.60)	(0.00)	(70.32)	(18.49)	(11.19)	(76.16)	(18.98)	(4.87)	(89.78)	(8.03)	(2.19)	(90.75)	(7.30)	(1.95)

⁎ Religion was not included; A, always; N, no; NS, not sure; NV, never; O, occasionally; Y, yes.

**Table 4:** Religious perspective and practice towards COVID-19 by demographic variables*

Characteristic		Religious perspective and practice, n (%)													
		R1: possiblity to pray and gather during the pandemic			R2: the place of worship conducted COVID-19 prevention control correctly			R3: social distancing in the place of worship make you feel safe from SARS-CoV-2 infection			RP1: pray in the place of worship other than home		RP2: how often did you pray and gather in the place of worship during “new normal”		
		Y	NS	N	Y	NS	N	Y	NS	N	Y	N	A	O	NV
Gender	Male	178	122	68	253	78	37	237	97	34	255	113	77	214	77
		(48.37)	(33.15)	(18.48)	(68.75)	(21.20)	(10.05)	(64.40)	(26.36)	(9.24)	(69.29)	(30.71)	(20.92)	(58.16)	(20.92)
	Female	280	466	240	648	227	111	629	289	68	278	708	46	360	580
		(28.40)	(47.26)	(24.34)	(65.72)	(23.02)	(11.26)	(63.79)	(29.31)	(6.90)	(28.19)	(71.81)	4.67	36.51	58.82
Age group	< 30	302	452	185	614	222	103	599	277	63	345	594	71	393	475
		(32.16)	(48.14)	(19.70)	(65.39)	(23.64)	(10.97)	(63.79)	(29.50)	(6.71)	(36.74)	(63.26)	(7.56)	(41.85)	(50.59)
	≥ 30	156	136	123	287	83	45	267	109	39	188	227	52	181	182
		(37.59)	(32.77)	(29.64)	(69.16)	(20.00)	(10.84)	(64.34)	(26.27)	(9.40)	(45.30)	(54.70)	(12.53)	(43.61)	(43.86)
Last education	High school	125	227	85	288	106	43	274	137	26	153	284	32	171	234
		(28.60)	(51.95)	(19.45)	(65.90)	(24.26)	(9.84)	(62.70)	(31.35)	(5.95)	(35.01)	(64.99)	(7.32)	(39.13)	(53.55)
	Associate	93	90	39	181	26	15	163	48	11	102	120	30	104	88
	degree	(41.89)	(40.54)	(17.57)	(81.53)	(11.71)	(6.76)	(73.42)	(21.62)	(4.95)	(45.95)	(54.05)	(13.51)	(46.85)	(39.64)
	Bachelor	169	211	119	304	123	72	314	143	42	190	309	40	219	240
	degree	(33.87)	(42.28)	(23.85)	(60.92)	(24.65)	(14.43)	(62.93)	(28.66)	(8.42)	(38.08)	(61.92)	(8.02)	(43.89)	(48.10)
	Master	53	50	46	99	35	15	87	46	16	65	84	17	58	74
	degree	(35.57)	(33.56)	(30.87)	(66.44)	(23.49)	(10.07)	(58.39)	(30.87)	(10.74)	(43.62)	(56.38)	(11.41)	(38.93)	(49.66)
	Doctoral	18	10	19	29	15	3	28	12	7	23	24	4	22	21
	degree	(38.30)	(21.28)	(40.43)	(61.70)	(31.91)	(6.38)	(59.57)	(25.53)	(14.89)	(48.94)	(51.06)	(8.51)	(46.81)	(44.68)
Major of education	Medicine related science	338	475	231	702	233	109	684	290	70	400	644	88	438	518
		(32.38)	(45.50)	(22.13)	(67.24)	(22.32)	(10.44)	(65.52)	(27.78)	(6.70)	(38.31)	(61.69)	(8.43)	(41.95)	(49.62)
	Science and technology	77	77	41	126	45	24	117	62	16	83	112	25	81	89
		(39.49)	(39.49)	(21.03)	(64.62)	(23.08)	(12.31)	(60.00)	(31.79)	(8.21)	(42.56)	(57.44)	(12.82)	(41.54)	(45.64)
	Social science and humanities	43	36	36	73	27	15	65	34	16	50	65	10	55	50
		(37.39)	(31.30)	(31.30)	(63.48)	(23.48)	(13.04)	(56.52)	(29.57)	(13.91)	(43.48)	(56.52)	(8.70)	(47.83)	(43.48)
Occupation	Students	200	340	124	429	170	65	424	204	36	239	425	46	262	356
		(30.12)	(51.20)	(18.68)	(64.61)	(25.60)	(9.79)	(63.86)	(30.72)	(5.42)	(35.99)	(64.01)	(6.93)	(39.46)	(53.61)
	Teacher	52	58	58	102	44	22	101	49	18	79	89	22	64	82
		(30.95)	(34.52)	(34.52)	(60.71)	(26.19)	(13.10)	(60.12)	(29.17)	(10.71)	(47.02)	(52.98)	(13.10)	(38.10)	(48.81)
	Health	151	142	89	289	60	33	259	94	29	161	221	45	185	152
	practitioners	(39.53)	(37.17)	(23.30)	(75.65)	(15.71)	(8.64)	(67.80)	(24.61)	(7.59)	(42.15)	(57.85)	(11.78)	(48.43)	(39.79)
	Government and administration related job	14	9	10	23	7	3	23	7	3	8	25	5	8	20
		(42.42)	(27.27)	(30.30)	(69.70)	(21.21)	(9.09)	(69.70)	(21.21)	(9.09)	(24.24)	(75.76)	(15.15)	(24.24)	(60.61)
	Others	41	39	27	58	24	25	59	32	16	46	61	5	55	47
		(38.32)	(36.45)	(25.23)	(54.21)	(22.43)	(23.36)	(55.14)	(29.91)	(14.95)	(42.99)	(57.01)	(4.67)	(51.40)	(43.93)
Place of current residence	City	293	412	238	647	207	89	602	267	74	346	597	87	354	502
		(31.07)	(43.69)	(25.24)	(68.61)	(21.95)	(9.44)	(63.84)	(28.31)	(7.85)	(36.69)	(63.31)	(9.23)	(37.54)	(53.23)
	Rural	165	176	70	254	98	59	264	119	28	187	224	36	220	155
		(40.15)	(42.82)	(17.03)	(61.80)	(23.84)	(14.36)	(64.23)	(28.95)	(6.81)	(45.50)	(54.50)	(8.76)	(53.53)	(37.71)

⁎ Religion was not included; A, always; N, no; NS, not sure; NV, never; O, occasionally; Y, yes.

**Table 5:** Results of multiple binary logistic regression analysis on factors significantly associated with attitudes, religious perspectives, and practice toward COVID-19

Variable	OR (95% CI)	P
Attitude and practice		
A1: not sure if physical distancing is able to cut the spread of SARS-CoV-2 (vs. yes)		
Gender (female vs. male)	0.60 (0.36 – 0.99)	0.047
Occupation (government and administration related job vs. teacher)	0.20 (0.08 – 0.49)	0.000
Occupation (government and administration related job vs. student)	0.19 (0.06 – 0.58)	0.003
Occupation (government and administration related job vs. other)	0.35 (0.15 – 0.81)	0.014
A2: not feeling anxious if being infected with SARS-CoV-2 (vs. yes)		
Gender (female vs. male)	0.54 (0.36 – 0.81)	0.003
Major of education (medicine related science vs. social science and humanities)	0.29 (0.12 – 0.69)	0.005
A2: not sure to feel anxious if being infected with SARS-CoV-2 (vs. yes)		
Last education (high school vs. associate degree)	0.29 (0.09 – 0.88)	0.029
Last education (high school vs. undergraduate degree)	0.26 (0.08 – 0.79)	0.018
Last education (high school vs. master degree)	0.28 (0.10 – 0.80)	0.017
Place of current residence (city vs. rural)	0.56 (0.41 – 0.78)	0.001
A3: large-scale social restriction need to be re-implemented (vs. no)		
Gender (female vs. male)	0.48 (0.30 – 0.78)	0.003
AP1: always performing COVID-19 prevention in a crowded place (vs. no)		
Gender (female vs. male)	0.30 (0.14 – 0.67)	0.003
AP1: always performing COVID-19 prevention in a crowded place (vs. occasionally)		
Gender (female vs. male)	0.45 (0.28 – 0.72)	0.001
Place of current residence (city vs. rural)	0.62 (0.39 – 0.99)	0.044
AP2: always performing COVID-19 prevention after traveling or working outside (vs. never)		
Gender (female vs. male)	0.32 (0.12 – 0.84)	0.020
AP2: always performing COVID-19 prevention after traveling or working outside (vs. occasionally)		
Gender (female vs. male)	0.43 (0.26 – 0.69)	0.001
Religious perspective and practice		
R1: it is not possible to pray and gather during the pandemic (vs. yes)		
Gender (female vs. male)	2.41 (1.70 – 3.42)	0.000
Place of current residence (city vs. rural)	1.84 (1.31 – 2.59)	0.000
R1: not sure whether it is possible to pray and gather during the pandemic (vs. yes)		
Gender (female vs. male)	2.15 (1.61 – 2.86)	0.000
Major of education (medicine related science vs. science and technology)	1.65 (1.00 – 2.73)	0.049
Place of current residence (city vs. rural)	1.38 (1.05 – 1.82)	0.020
R2: the place of worship is not conducted COVID-19 prevention control correctly (vs. yes)		
Last education (high school vs. master degree)	4.49 (1.11 – 18.20)	0.035
Occupation (government and administration related job vs. teacher)	0.26 (0.13 – 0.49)	0.000
Occupation (government and administration related job vs. other)	0.28 (0.14 – 0.55)	0.000
Place of current residence (city vs. rural)	0.56 (0.39 – 0.82)	0.003
R2: not sure whether the place of worship conducted COVID-19 prevention control correctly (vs. yes)		
Last education (high school vs. undergraduate degree)	0.31 (0.12 – 0.79)	0.014
Occupation (government and administration related job vs. teacher)	0.53 (0.29 – 0.97)	0.039
R3: social distancing in the place of worship make you feel safe from SARS-CoV-2 infection (vs. no)		
Occupation (government and administration related job vs. others)	0.26 (0.11 – 0.62)	0.002
RP1: not going to pray in the place of worship other than home (vs. yes)		
Gender (female vs. male)	5.88 (4.48 – 7.72)	0.000
Occupation (government and administration related job vs. health practitioners)	2.78 (1.06 – 7.24)	0.037
Place of current residence (city vs. rural)	1.54 (1.19 – 1.99)	0.001
RP2: never going to pray and gather in the place of worship during “new normal” (vs. always)		
Gender (female vs. male)	13.75 (8.64 – 21.87)	0.000
Occupation (government and administration related job vs. teacher)	0.15 (0.03 – 0.65)	0.012
RP2: never going to pray and gather in the place of worship during “new normal” (vs. occasionally)		
Gender (female vs. male)	4.52 (3.33 – 6.14)	0.000
Occupation (government and administration related job vs. health practitioners)	2.74 (1.06 – 7.08)	0.038
Place of current residence (city vs. rural)	2.11 (1.63 – 2.75)	0.000

## 2 Experimental Design, Materials and Methods

### 2.1 Participants

This cross-sectional survey was conducted from July 11 – 18, 2020. Data collection relied on the authors’ network; one-page recruitment information was posted/reposted via Whatsapp. This information contained a brief introduction about the survey, voluntary nature of participation, declarations of anonymity and confidentiality, and the link of the online questionnaire. Persons who were of Indonesian nationality, aged 16 years or more, and willing to participate were directed to complete the survey.

### 2.2 Measures

The questionnaire consisted of three parts: demographics, attitudes and practices, and religious perspectives and practices. Demographic variables included age, gender, last education, major of education or current education, occupation, place of current residence (city vs. rural), and religion. Attitudes and practices toward COVID-19 were evaluated by questions A1 – A3 and AP1 – 2 (Table 1), while religious perspectives and practices were measured by questions R1 – R3 and RP1 – 2 (Table 1).

### 2.3 Statistical analysis

Frequencies of attitudes, religious perspectives, and practices were tabulated. Attitudes, religious perspectives, and practices of different persons according to demographic characteristics (excluding religion) were compared with the Chi-square test. The binary logistic regression method was used to identify factors associated with attitudes, religious perspectives, and practices. Data analyses were conducted with SPSS version 25 for Mac. A p-value of less than 0.05 (two-sided) was considered significant.

### 2.4 Ethical statement

This survey was approved by the Ethics Committee of Brawijaya University, Malang-Indonesia, with reference No. 062-KEP-UB-2020.

## Funding

This survey did not receive any specific grant from funding agencies in the public, commercial, or not-for-profit sectors

## Declaration of Competing Interest

None to declare.

## Appendix Supplementary materials

Image, application 1
